# The Impact of Virtual Cancer Care on Chemotherapy Delivery and Clinical Outcomes in Colorectal Cancer Patients Receiving Systemic Therapy: A Pre- and Intra-Pandemic Analysis

**DOI:** 10.3390/curroncol29090489

**Published:** 2022-08-29

**Authors:** William J. Phillips, Macyn Leung, Kednapa Thavorn, Timothy R. Asmis

**Affiliations:** 1 Faculty of Medicine, University of Ottawa, Ottawa, ON K1N 6N5, Canada; 2 The Ottawa Hospital Research Institute, Ottawa, ON K1H 8L6, Canada; 3 School of Epidemiology and Public Health, University of Ottawa, Ottawa, ON K1N 6N5, Canada; 4 The Ottawa Hospital Cancer Centre, Ottawa, ON K1H 8L6, Canada

**Keywords:** virtual care, colorectal cancer, chemotherapy, COVID-19

## Abstract

(1) Background: The coronavirus 2019 pandemic has resulted in an abrupt transition to virtual oncology care worldwide. This study’s objective is to evaluate chemotherapy delivery and clinical outcomes in patients on systemic treatment for colorectal cancer before and during the pandemic. (2) Methods: Clinical data was collected on patients with colorectal cancer receiving intravenous chemotherapy at The Ottawa Hospital from June 2019 to March 2021. Patients were stratified by whether they were started on chemotherapy pre-pandemic (June 2019–January 2020) or intra-pandemic (February 2020–March 2021). Multiple regression analysis was used to compare outcomes between pandemic periods; (3) Results: There were 220 patients included in this study. The proportion of virtual consultations (1.2% to 64.4%) and follow-up visits (5.2% to 83.3%) increased during the pandemic. There was no difference in the incidence of treatment delays (OR = 1.01, *p* = 0.78), chemotherapy dose reductions (OR = 0.99, *p* = 0.69), emergency department visits (OR = 1.23, *p* = 0.37) or hospitalizations (OR = 0.73, *p* = 0.43) between pandemic periods. A subgroup analysis revealed no difference in outcomes independent of the presence of metastases; (4) Conclusion: These findings serve as an important quality-care indicator and demonstrate that virtual oncology care appears safe in a cohort of high-risk colorectal cancer patients.

## 1. Introduction

Colorectal cancer is the third most common cancer worldwide and second in Canada [1]. Multidrug chemotherapy regimens are widely used for treatment in both the adjuvant and metastatic setting. Fluoropyrimidine-based agents are the backbone of therapy and commonly used systemic treatment combinations include folinic acid/fluorouracil/oxaliplatin (FOLFOX), folinic acid/fluorouracil/irinotecan (FOLFIRI) or capecitabine/oxaliplatin (CAPOX) with or without monoclonal antibodies [2,3,4,5]. Although the routine uses of combination therapy is responsible for improved patient outcomes, it is also carries a significant risk of treatment related toxicity that can necessitate adjustment to the original chemotherapy plan to allow for recovery [6]. Treatment delays and dose reductions are common modifications. In severe cases, chemotherapy can be discontinued entirely, particularly in the adjuvant setting. Ideally, clinicians aim to provide the highest relative dose intensity possible to replicate the treatments received in clinical trials and optimize patient outcomes [7,8,9]. The delivery of full dose intensity chemotherapy is considered a quality-of-care indicator in oncology [10].

Traditionally, patients are seen regularly in clinic by their oncology team to screen for treatment related side effects and facilitate early management of chemotherapy toxicities. The emergence of coronavirus disease 2019 (COVID-19) resulted in an unprecedent shift to virtual cancer care, including patients on active systemic therapy. This decision was based due to the high risk of complications related to COVID-19 infection [11]. Virtual care can be defined as any interaction between clinician and patient that is not performed in-person, primarily by video conferencing or telephone. In most cancer centers worldwide, the shift to virtual care was abrupt and took place over a matter of days [12].

In this study, we leverage the rapid adoption of virtual oncology care during the COVID-19 pandemic to evaluate its impact on chemotherapy delivery and clinical outcomes. We chose to study a cohort of colorectal cancer patients on active treatment with intravenous (IV) chemotherapy agents to assess the safety of virtual care in patients at high-risk of treatment related adverse events.

## 2. Methods

### 2.1. Study Design

We conducted a retrospective cohort study of patients diagnosed with colorectal cancer at The Ottawa Hospital Cancer Centre consecutively between 1 June 2019, and 31 March 2021 (n = 1,573) and treated with IV chemotherapy (n = 282). We excluded patients with stage 1–3 rectal cancer (n = 220), due to the various treatment options and multidisciplinary nature of treatment selection in this population.

We defined the pandemic period according to the date of chemotherapy initiation. Patients beginning chemotherapy from 1 June 2019 to 31 January 2020 were categorized as “pre-pandemic”, while patients beginning from 1 February 2020 to 31 March 2021 were categorized as “intra-pandemic”. Our center declared a public health state of emergency and shifted towards virtual care 22 March 2020. We selected 1 February 2020 as the pandemic period cut-off date to provide a buffer for patients started on chemotherapy just before the public health emergency declaration. Patients were followed until the discontinuation of first line chemotherapy or the end of the observation period (30 September 2021); outcomes that occurred after this point were censored.

### 2.2. Patient Characteristics

All patient information was gathered from the Ottawa Hospital’s electronic medical records (EMR), including clinical notes, oncology treatment plans and pathology/radiology reports. Baseline characteristics including age, gender, functional status, body mass index and health comorbidities, as estimated by the modified Charlson Comorbidity Index, were collected. The Charlson Comorbidity Index was modified to omit points for the presence of malignancy [13]. Cancer characteristics such as stage, histology and molecular markers were also collected.

All cancer treatment data from The Ottawa Hospital Cancer Centre was available on the local EMR, regardless of whether chemotherapy was delivered at a satellite campus. The Ottawa Hospital Cancer Centre is the regional cancer center for its entire local health network and encompasses several satellites treatment sites where chemotherapy is delivered using the same EMR. Treatment data included chemotherapy regimen, initial dose, frequency of cycles, number of cycles planned, number of cycles received, and receipt of targeted therapies. Visit type was gathered from Ottawa Hospital administrative databases. Each visit was given a specific code depending on its type (consultation versus follow-up) and method of assessment (in-person versus virtual). Consultations were defined as the first outpatient assessment after diagnosis, while follow-ups were defined as any subsequent visit. Virtual visits consisted of telephone encounters and electronic video conferencing; these were grouped together for the purposes of analysis. Consultation data was not available after June 2021, based on when the health records department pulled the data for this study.

### 2.3. Defining Outcomes of Interest

Outcomes of interest were divided into chemotherapy delivery and clinical outcomes. Chemotherapy delivery outcomes included treatment delays, dose reductions and early discontinuations. Treatment delays were considered if a chemotherapy cycle was delayed ≥1 week from the planned treatment date, dose reduction if a chemotherapy cycle’s dose was reduced by ≥10% and early discontinuation if intravenous chemotherapy was terminated prior to the planned stop date. Early discontinuations were only captured in patients undergoing adjuvant therapy, where there was a prespecified number of cycles. Clinical outcomes were composed of emergency department (ED) visits and hospitalizations. These were collected by review of oncology clinical notes, records of emergency assessment or admission notes at the Ottawa Hospital. ED visits to select community hospitals that share an EMR with the Ottawa Hospital were available as well. No survival outcomes were analyzed.

### 2.4. Statistical Analysis

Descriptive analyses were used to describe baseline and treatment characteristics of the study population. Statistical analysis was stratified by pandemic period: pre-pandemic vs. intra-pandemic, which are defined above. The relationship between categorical variables was assessed using the Fischer’s Exact Test, while continuous variables were assessed using the Mann–Whitney U test. A binary logistic regression was used to evaluate the relationship between pandemic period and the outcomes of interest for binary variables, while generalized linear regression with a negative binomial (log linked) distribution was used for continuous variables. A subgroup analysis was performed based on the presence of metastatic disease. SPSS (IBM Corp. Released 2021. IBM SPSS Statistics for Mac, Version 28.0. Armonk, NY, USA) was used for statistical analysis. Microsoft Excel (Microsoft Corp. Released 2021. Microsoft Excel for Mac, Version 16.54) and GraphPad Prism 9 (GraphPad Prism Version 9.3.1 (350) for macOS, released December 7, 2021. Sourced from San Diego, CA, USA) were used to display the results in tables and figures. Confidence intervals were represented as 95% certain to contain the population mean. A *p*-value of < 0.05 was considered the threshold of statistical significance.

## 3. Results

### 3.1. Sample Characteristics

Baseline characteristics of the study participants are summarized in Table 1. There were 220 patients with colorectal cancer treated with IV chemotherapy included in this study. The mean age was 61 years (standard deviation (SD) = 11.0) and there were 102 (46.4%) females. The most common chemotherapy regimen was folinic acid/fluorouracil/oxaliplatin (FOLFOX, 47.7%), followed by capecitabine/oxaliplatin (CAPOX, 25.5%) and folinic acid/fluorouracil/oxaliplatin (FOLFIRI, 22.7%). Patients received 9.6 cycles of chemotherapy on average. There were 16 (10.6%) patients found to have microsatellite instability. Patients in the intra-pandemic period had a lower mean modified Charlson co-morbidity index (2.4 vs.1.9, *p* = 0.041). A trend towards increased CAPOX receipt during the pandemic was also observed (pre-pandemic: n = 10, (15.2%) versus intra-pandemic: n = 46 (29.9%)). Otherwise, there were no significant differences in baseline characteristics between pandemic periods.

### 3.2. Virtual Consultation and Follow-Up Visits

**Figure 1:**
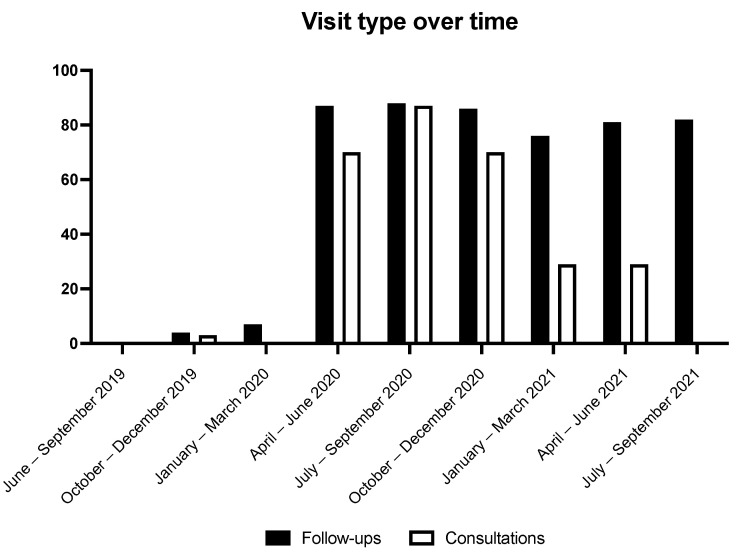
The relationship of visit type over time during the observation period. The Y-axis represents the proportion of virtual visits.

Patients were more likely to receive virtual care during the pandemic. Of the 85 consultations performed before the pandemic, 1 (1.2%) was virtual compared to 87 of 135 (64.4%) during the pandemic. Likewise, of the 463 follow-up visits performed before the pandemic, 24 (5.2%) were virtual compared to 1513 of 1891 (83.3%) during the pandemic. This represents a relative increase of 63.2 and 77.9% for virtual consultations and follow-up visits during the pandemic, respectively. It is important to note that no consultation data was available from July to September 2021. The trend of virtual care over time was evaluated, see Figure 1. The proportion of virtual follow-up visits appears stable throughout the pandemic period, while rates of virtual consultations increase early in the pandemic and fall over time.

### 3.3. Chemotherapy Continuity and Clinical Outcomes

Treatment delays (128, 58.2%) were the most common outcome observed in our population, followed by dose reductions (116, 52.7%) and hospitalizations (39, 17.7%). On average, patients experienced 0.46 (SD = 0.92) ED visits per treatment course.

The relationship of outcomes and pandemic period were evaluated, see Table 2. There were no significant differences in the incidence of chemotherapy delays (odds ratio (OR) = 1.11, *p* = 0.75), dose reductions (OR = 1.19, *p* = 0.58) or hospitalizations (OR = 0.72, p = 0.39) between patients receiving IV chemotherapy in the pre-pandemic versus intra-pandemic period. Similarly, we did not find significant associations of the pandemic period and the mean number (rate ratio (RR) = 1.20, *p* = 0.39) or the cumulative length of treatment delays (RR = 1.25, *p* = 0.26). The number of ED visits over the course of treatment (RR = 0.97, *p* = 0.90) was similar between pandemic periods, as well. These analyses were controlled for age, chemotherapy type, modified Charlson comorbidity index, metastatic status and number of cycles received.

### 3.4. Analysis of Outcomes Based on the Presence of Metastatic Disease

**Figure 2:**
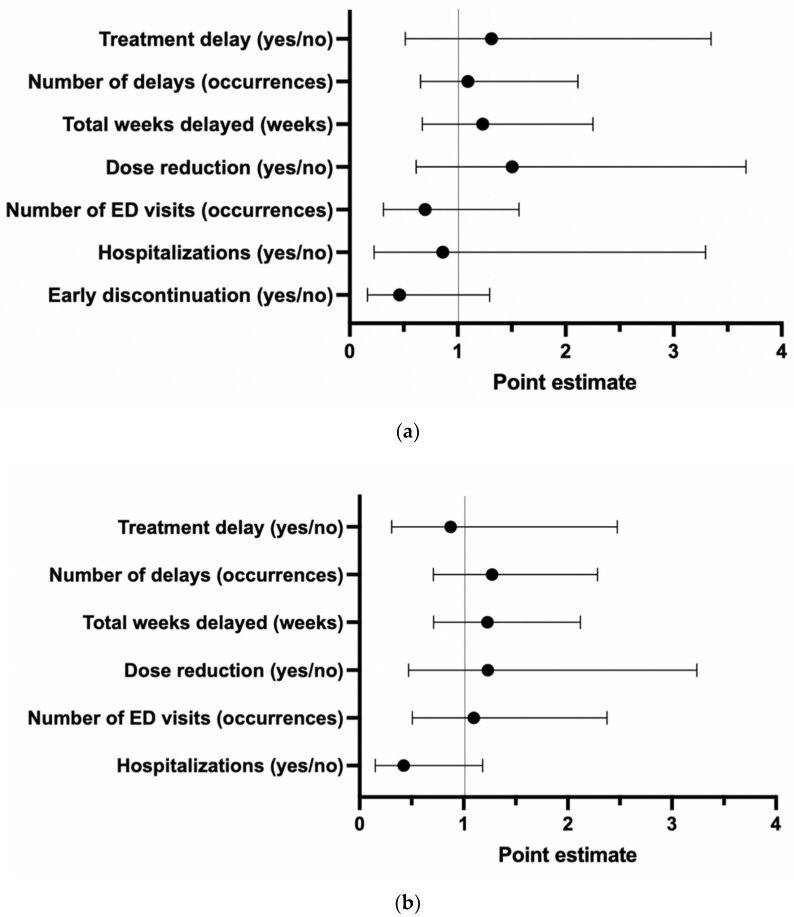
Subgroup analysis of patients with (**a**) nonmetastatic and (**b**) metastatic colorectal cancer represented as a Forest Plot of the point estimates and 95% confidence intervals for each outcome compared in the pre- versus intra-pandemic period, where a positive estimate indicates that the outcome is more likely in the intra-pandemic period. Models adjusted for age, chemotherapy type, modified Charlson comorbidity index, and number of cycles received.

There were 112 patients with local disease and 108 with metastatic disease. The most common site of metastatic disease was the liver (68.5%), followed by peritoneum (30.6%), lung (17.6%) and bone (4.6%). There were no significant differences in the incidence of treatment delays, dose reductions or hospitalizations between pandemic periods in patients with nonmetastatic and metastatic disease. There were also no significant differences in the mean number of treatment delays, cumulative length of treatment delays or number of ED visits in either subgroup. The relationship of clinical outcomes stratified by the presence of metastatic disease is summarized in Figure 2.

## 4. Discussion

Virtual encounters have been integral in delivering oncology care to patients during the COVID-19 pandemic. At most Canadian centers, virtual cancer care remains a principal method of assessment. The data presented in this article demonstrates an abrupt transition to virtual oncology care in April 2020 and sustained use over 1 year after the pandemic began. Follow-up visits remained predominantly virtual throughout the study period, while the rate of virtual consultations decreased later in the pandemic. It is commonly believed that initial care plan discussions should be performed in-person, which may explain why in-person consultations became more common later in the pandemic [14]. Furthermore, this study shows no significant difference in chemotherapy delivery or clinical outcomes in patients started on treatment during the pandemic. This serves as an important quality assurance measure demonstrating the safety of virtual oncology care during the COVID-19 pandemic, in patient undergoing active treatment for colorectal cancer.

Virtual cancer care was first introduced in Canada, albeit in a limited capacity, in 2003 [15]. Until the COVID-19 pandemic, virtual cancer care was being used sparsely even for residents living in remote geographies. In Ontario, problems with physician reimbursement and the mandated use of select, pre-approved video conferencing technology limited the role of virtual care in clinical practice [16]. These barriers were eliminated during the first wave of the COVID-19 pandemic, where Ontario Health Insurance Plan (OHIP) developed temporary billing codes for virtual care delivered via telephone or mainstream web conferencing software (i.e., Zoom, Skype, etc.) making virtual care practical for clinicians. Recently, an Ontario-wide consensus statement was released to guide virtual oncology care using a modified Delphi of 29 interdisciplinary panel members, demonstrating that momentum is beginning to be gained in virtual oncology care in Canada [17].

Despite being a pillar of clinical assessment throughout the pandemic, virtual cancer care is appealing beyond pandemic circumstances too. It offers the possibility of more accessible care to patients residing in remote geographies, including patients residing in Northern Canadian communities that often have barriers to cancer care access [18]. It is also convenient, reduces travel time and lowers the cost associated with accessing care for patients [19,20]. On a system level, virtual care has the potential of providing safe, efficient and cost-effective follow-up and survivorship care to a rapidly growing Canadian cancer population [21,22]. Canadian data suggests that both patients and clinicians are generally satisfied with virtual care [23]. Similar levels of satisfaction with virtual care have been observed internationally, as well [24,25].

Current evidence for virtual oncology care is strongest for low-risk follow-up and survivorship care [26]. This has led to debate regarding how virtual care compares to traditional in-person assessment for patients undergoing active treatment. An Australian study compared 89 patients undergoing chemotherapy receiving virtual follow-up to 117 patients followed in-person. They concluded no differences in the incidence of adverse effects of therapy or hospital admissions between the two groups [27]. A separate study, evaluating the safety and cost savings of telemedicine during the pandemic in a cohort of cancer patients receiving intravenous treatment, reported no differences in clinical outcomes over a 3-month observation period [20]. Our study adds to this data by reproducing safety outcomes in a population of colorectal cancer patients receiving, predominantly, combination chemotherapy. In addition, patients with metastatic disease are typically at higher risk for toxicity based on the higher cumulative dose of chemotherapy received and larger tumor burdens. The subgroup analysis presented above shows comparable chemotherapy delivery outcomes in patients with metastatic disease, suggesting that virtual oncology care appears safe even in a high-risk cohort of colorectal cancer patients.

There continues to be barriers impacting widespread use of virtual care into clinical practice. For instance, triaging patients who are appropriate for virtual assessment remains a challenge. Survey data suggests that health-care providers feel that there are inadequate guidelines available to distinguish when a patient requires an in-clinic assessment versus when they are safe for virtual care [28]. Serious illness discussion and palliative care are other areas where health-care providers feel less comfortable with a virtual format [26]. Currently, most virtual cancer care is being performed via telephone rather than platforms that allow video-conferencing in Canada [28,29]. The regular use of video-conferencing software may help bridge this gap by enhancing nonverbal communication thus improving the connection between clinician and patient during sensitive discussions. Other obstacles that need to addressed include privacy concerns, lack of technology expertise of patients and lack of physical examination [30]. Finally, implementation of virtual nursing care is an important consideration. Nurses working in oncology clinics play a major role in supporting clinicians with patient education, treatment counselling, patient assessment and caring for the psychological, emotional, and social aspects of cancer [31]. A standardized framework for delivering virtual oncology nursing care will need to be explored to prevent marginalization of their role.

This work may lack applicability to other virtual care models, as a single-centered retrospective study. That said, The Ottawa Hospital Cancer Centre is one of Canada’s largest cancer centers. It has a catchment area of approximately 1.4 million people and operates multiple satellite chemotherapy units, as well as providing remote care to patients in the Baffin region of Nunavut. This study may also be limited by its sample size, which may have impacted the ability to detect small differences in outcomes between pandemic periods, particularly when stratifying by the presence of metastasis. There were restrictions in data availability outside of our EMR. The EMR used at the Ottawa Hospital Cancer Centre encompasses all oncology satellite treatment centers, therefore treatment quality outcomes should be captured in full. Many local community hospitals share an EMR with our center but patients who sought urgent medical attention outside of the network may have been missed. Furthermore, there was tendency towards differential use of chemotherapy regiments during the pandemic. For instance, CAPOX use approximately doubled in the pandemic period, which may reflect the results of the IDEA collaboration meta-analysis demonstrating non-inferiority of 3 months CAPOX to traditional 6 months of adjuvant therapy in the subgroup analysis or the emphasis on limiting addition chemotherapy chair time [32]. CAPOX is delivered in 3-week cycles compared to FOLFOX, which is delivered in 2-week cycles. This may also explain the tendency towards less cycles of chemotherapy being delivered in the pandemic period. Finally, virtual care is one of many factors impacted by the COVID-19 pandemic. Factors such as public health regulations, fear of infection, impeded access to non-oncology health services and COVID-19 infection were outside the scope of this study. In particular, fear of infection and hospital avoidance may explain why there a trend towards less hospitalization in the pandemic era.

## 5. Conclusions

Overall, this study demonstrates no significant differences in chemotherapy delivery, emergency department use or hospitalizations in patients with colorectal cancer treated with IV chemotherapy during the COVID-19 pandemic. These findings serve as an important quality-care indicator and demonstrate that virtual oncology care appears safe in a cohort of high-risk colorectal cancer patients receiving systemic therapy. Future work dedicated to other tumor sites and cancer centers would allow for broader application of these findings.

## Figures and Tables

**Table 1:** Baseline characteristics of study population.

Characteristic	Overall (n = 220)	Pre-Pandemic (n = 66)	Intra-Pandemic (n = 154)	*p*-Value
Age (mean, SD)	61 (11)	63.1 (11.6)	60.1 (10.6)	0.055
Sex (female)	102 (46.4%)	32 (48.5%)	70 (45.5)	0.680
Body mass index (mean, SD)	28 (7.1)	26.8 (6.1)	28.5 (7.6)	0.061
Modified Charlson index (mean, SD)	2.1 (1.4)	2.4 (1.4)	1.9 (1.3)	0.041
Presence of metastatic disease	108 (49.1%)	32 (48.5%)	78 (50.6%)	0.91
Location of primary (n = 217)				
Right	88 (40.5%)	29 (45.3%)	59 (38.6%)	
Left	108 (49.8%)	31 (48.4%)	77 (50.3%)	
Rectal	21 (9.7%)	4 (6.3%)	17 (11.1%)	
Microsatellite instability (n = 151)	16 (10.6%)	8 (36.4%)	8 (7.1%)	
NRAS or KRAS mutated (n = 91)	30 (33%)	9 (40.9%)	21 (30.4%)	
BRAF mutated (n = 91)	17 (18.7%)	5 (22.7%)	12 (17.4%)	
Chemotherapy Type				0.091
*CAPOX*	56 (25.5%)	10 (15.2%)	46 (29.9%)	
*FOLFOX*	105 (47.7%)	38 (57.6%)	67 (43.5%)	
*FOLFIRI*	50 (22.7%)	17 (25.8%)	33 (21.4%)	
*FOLFIRINOX*	6 (2.7%)	1 (1.5%)	5 (3.2%)	
*Other*	3 (1.4%)	0 (0%)	3 (1.9%)	
Receipt of MOA				0.24
*VEGF*	47 (21.4%)	18 (27.3%)	29 (18.8%)	
*EGFR*	8 (3.6%)	1 (1.5%)	7 (4.5%)	
*None*	165 (75%)	47 (71.2%)	118 (76.6%)	
Number of cycles (mean, SD)	8.7 (6.0)	10.8 (8.1)	7.8 (4.6)	0.051

Abbreviations: SD, standard deviation; CAPOX, capecitabine/oxaliplatin; FOLFOX, folinic acid/fluorouracil/oxaliplatin; FOLFIRI folinic acid/fluorouracil/irinotecan; FOLFIRINOX, folinic acid/fluorouracil/irinotecan/oxaliplatin; MOA, monoclonal antibody. Significance statistics not calculated for fields with missing values.

**Table 2:** The relationship of starting chemotherapy during the COVID-19 pandemic on treatment interruptions, ED use and hospitalizations by multiple regression analysis adjusted for age, chemotherapy type, modified Charlson comorbidity index, metastatic status and number of cycles received.

Outcome	Relative Frequency (Pre/Intra)	Adjusted Point Estimate (95% CI)	*p*-Value
Presence of a treatment delay	60.6%/57.1%	1.11 (0.58–2.15) *	0.75
Number of treatment delays (mean)	1.27/1.21	1.20 (0.79–1.84) †	0.39
Cumulative length of delay (mean weeks)	2.24/1.92	1.25 (0.85–1.85) †	0.26
Presence of a dose reduction	53.0%/52.6%	1.19 (0.64–2.21) *	0.58
Number of ED visits (mean)	0.48/0.45	0.97 (0.56–1.66) †	0.90
Hospitalization	22.7%/15.6%	0.72 (0.33–1.54) *	0.39

Point estimates were represented as odds ratios for binary outcomes (*) and rate ratios (†) for continuous data. (Abbreviations: ED, emergency department; CI, confidence interval; pre, pre-pandemic period; intra, intra-pandemic period).

## Data Availability

The data presented in this study are available on reasonable request from the corresponding author. The data are not publicly available due to restrictions in place to protect patient privacy.

## References

[1] Canadian Cancer Statistics Advisory Committee (2021). Canadian Cancer Statistics.

[2] Loupakis F., Cremolini C., Masi G., Lonardi S., Zagonel V., Salvatore L., Cortesi E., Tomasello G., Ronzoni M., Spadi R. (2014). Initial Therapy with FOLFOXIRI and Bevacizumab for Metastatic Colorectal Cancer. N. Engl. J. Med..

[3] Saltz L.B., Clarke S., Díaz-Rubio E., Scheithauer W., Figer A., Wong R., Koski S., Lichnitser M., Yang T.S., Rivera F. (2008). Bevacizumab in combination with oxaliplatin-based chemotherapy as first-line therapy in metastatic colorectal cancer: A randomized phase III study. J. Clin. Oncol..

[4] Aparicio T. (2011). Oxaliplatin, fluorouracil and leucovorin as adjuvant treatment for colon cancer. Colon Rectum.

[5] Kelly H., Goldberg R.M. (2005). Systemic therapy for metastatic colorectal cancer: Current options, current evidence. J. Clin. Oncol..

[6] Aoullay Z., Slaoui M., Razine R., Er-Raki A., Meddah B., Cherrah Y. (2020). Therapeutic Characteristics, Chemotherapy-Related Toxicities and Survivorship in Colorectal Cancer Patients. Ethiop. J. Health Sci..

[7] Denduluri N., Patt D.A., Wang Y., Bhor M., Li X., Favret A.M., Morrow P.K., Barron R.L., Asmar L., Saravanan S. (2015). Dose delays, dose reductions, and relative dose intensity in patients with cancer who received adjuvant or neoadjuvant chemotherapy in community oncology practices. JNCCN J. Natl. Compr. Cancer. Netw..

[8] Havrilesky L.J., Reiner M., Morrow P.K., Watson H., Crawford J. (2015). A review of relative dose intensity and survival in patients with metastatic solid tumors. Crit. Rev. Oncol. Hematol..

[9] Chang J. (2000). Chemotherapy dose reduction and delay in clinical practiceevaluating the risk to patient outcome in adjuvant chemotherapy for breast cancer. Eur. J. Cancer..

[10] Lyman G.H. (2009). Impact of chemotherapy dose intensity on cancer patient outcomes. JNCCN J. Natl. Compr. Cancer. Netw..

[11] Liang W., Guan W., Chen R., Wang W., Li J., Xu K., Li C., Ai Q., Lu W., Liang H. (2020). Cancer patients in SARS-CoV-2 infection: A nationwide analysis in China. Lancet. Oncol..

[12] Rodin D., Lovas M., Berlin A. (2020). The reality of virtual care: Implications for cancer care beyond the pandemic. Healthc..

[13] Asmis T.R., Ding K., Seymour L., Shepherd F.A., Leighl N.B., Winton T.L., Whitehead M., Spaans J.N., Graham B.C., Goss G.D. (2008). Age and comorbidity as independent prognostic factors in the treatment of non-small-cell lung cancer: A review of National Cancer Institute of Canada Clinical Trials Group Trials. J. Clin. Oncol..

[14] Liu R., Sundaresan T., Reed M.E., Trosman J.R., Weldon C.B., Kolevska T. (2020). Telehealth in Oncology During the COVID-19 Outbreak: Bringing the House Call Back Virtually. JCO. Oncol. Pract..

[15] Palkhivala A. (2011). Canada develops models of teleoncology. J. Natl. Cancer Inst..

[16] Bhatia R.S., Chu C., Pang A., Tadrous M., Stamenova V., Cram P. (2021). Virtual care use before and during the COVID-19 pandemic: A repeated cross-sectional study. CMAJ Open..

[17] Cheung M.C., Franco B.B., Meti N., Thawer A., Tahmasebi H., Shankar A., Loblaw A., Wright F.C., Fox C., Peek N. (2021). Delivery of Virtual Care in Oncology: Province-Wide Interprofessional Consensus Statements Using a Modified Delphi Process. Curr. Oncol..

[18] Febbraro M., Conlon M., Caswell J., Laferriere N. (2020). Access to cancer care in Northwestern Ontario—A population-based study using administrative data. Curr. Oncol..

[19] Elkaddoum R., Haddad F.G., Eid R., Kourie H.R. (2020). Telemedicine for cancer patients during COVID-19 pandemic: Between threats and opportunities. Futur. Oncol..

[20] Hsiehchen D., Muquith M., Haque W., Espinoza M., Yopp A., Beg M.S. (2021). Clinical Efficiency and Safety Outcomes of Virtual Care for Oncology Patients During the COVID-19 Pandemic. JCO Oncol. Pract..

[21] Pham Q., Hearn J., Bender J.L., Berlin A., Brown I., Bryant-Lukosius D., Feifer A.H., Finelli A., Gotto G., Hamilton R. (2021). Virtual care for prostate cancer survivorship: Protocol for an evaluation of a nurse-led algorithm-enhanced virtual clinic implemented at five cancer centres across Canada. BMJ Open..

[22] Pham Q., Hearn J., Gao B., Brown I., Hamilton R.J., Berlin A., Cafazzo J.A., Feifer A. (2020). Virtual care models for cancer survivorship. NPJ Digit. Med.

[23] Berlin A., Lovas M., Truong T., Melwani S., Liu J., Liu Z.A., Badzynski A., Carpenter M.B., Virtanen C., Morley L. (2021). Implementation and Outcomes of Virtual Care Across a Tertiary Cancer Center during COVID. JAMA Oncol..

[24] Loree J.M., Dau H., Rebić N., Howren A., Gastonguay L., McTaggart-Cowan H., Gill S., Raghav K., De Vera M.A. (2021). Virtual oncology appointments during the initial wave of the COVID-19 pandemic: An international survey of patient perspectives. Curr. Oncol..

[25] Orlando J.F., Beard M., Kumar S. (2019). Systematic review of patient and caregivers’ satisfaction with telehealth videoconferencing as a mode of service delivery in managing patients’ health. PloS ONE.

[26] Singh S., Fletcher G.G., Yao X., Sussman J. (2021). Virtual care in patients with cancer: A systematic review. Curr Oncol..

[27] Chan B.A., Larkins S.L., Evans R., Watt K., Sabesan S. (2015). Do teleoncology models of care enable safe delivery of chemotherapy in rural towns?. Med. J. Aust..

[28] Watson L., Qi S., Delure A., Link C., Photitai E., Chmielewski L., Hildebrand A., Ruether D., Rawson K. (2021). Virtual Cancer Care During the COVID-19 Pandemic in Alberta: Evidence From a Mixed Methods Evaluation and Key Learnings. JCO Oncol. Pract..

[29] Levine O.H., McGillion M., Levine M. (2020). Virtual cancer care during the COVID-19 pandemic and beyond: A call for evaluation. JMIR Cancer.

[30] Mussetti A., Peric Z., Figueroa C. (2022). COVID19 in hematological patients and telemedicine: Lessons learned across Europe and the US. Curr. Opin. Infect. Dis..

[31] Charalambous A. (2020). Specialized Cancer Care Roles: From Clinical Practice to Research and beyond. Asia-Pacific J. Oncol. Nurs..

[32] Grothey A., Sobrero A.F., Shields A.F., Yoshino T., Paul J., Taieb J., Souglakos J., Shi K., Kerr R., Labianca R. (2018). Duration of Adjuvant Chemotherapy for Stage III Colon Cancer. N. Engl. J. Med..

